# Estimating temperature-dependent anisotropic hydrogen displacements with the invariom database and a new segmented rigid-body analysis program

**DOI:** 10.1107/S1600576715018075

**Published:** 2015-11-10

**Authors:** Jens Lübben, Luc J. Bourhis, Birger Dittrich

**Affiliations:** a Institut für Anorganische und Angewandte Chemie, Martin-Luther-King-Platz 6, 20146 Hamburg, Germany; b Bruker-AXS SAS, 4 Allée Lorents, F-77447 Marne-la-Vallée, France; c Heinrich-Heine Universität Düsseldorf, Anorganische Chemie und Strukturchemie, Universitätsstrasse 1, Gebäude 26.42.01.21, 40225 Düsseldorf, Germany

**Keywords:** anisotropic displacement parameters, generalized invariom database, segmented rigid-body analysis

## Abstract

Invariom partitioning and notation are used to estimate anisotropic hydrogen displacements for incorporation in crystallographic refinement models. Optimized structures of the generalized invariom database and their frequency computations provide the information required: frequencies are converted to internal atomic displacements and combined with the results of a TLS (translation–libration–screw) fit of experimental non-hydrogen anisotropic displacement parameters to estimate those of H atoms. Comparison with TLS+ONIOM and neutron diffraction results for four example structures where high-resolution X-ray and neutron data are available show that electron density transferability rules established in the invariom approach are also suitable for streamlining the transfer of atomic vibrations. A new segmented-body TLS analysis program called *APD-Toolkit* has been coded to overcome technical limitations of the established program *THMA*. The influence of incorporating hydrogen anisotropic displacement parameters on conventional refinement is assessed.

## Abstract 2

A novel method and a new program for estimating anisotropic displacement parameters for H atoms are presented. Results are validated against molecular orbital computations and neutron diffraction data.

## 1. Introduction   

Improving the accuracy of structural information derived from conventional single-crystal X-ray diffraction (XRD) experiments has been the initial aim for transferring aspherical scattering factors (Brock *et al.*, 1991 ▸) and it remains the central aim of the generalized invariom database (GID; Dittrich *et al.*, 2013 ▸). Deriving molecular properties from aspherical electron density is another important and closely related aim, since such properties can only be as accurate as the best possible set of coordinates that can be refined from a given data set.

Anisotropic displacement parameters (ADPs) of atoms and their electron density distribution (EDD) are correlated  (Hirshfeld, 1976 ▸). It has hence been shown to be beneficial to incorporate hydrogen ADPs for obtaining accurate EDDs by least-squares refinement of multipole parameters against high-resolution data  (Madsen *et al.*, 2004 ▸). Since this must also hold true for parameters derived from conventional data sets, our recent efforts concern the estimation of temperature-dependent hydrogen ADPs (H-ADPs) for selected applications in structure determinations with the independent atom model (IAM) as well as refinement with aspherical scattering factors. This includes charge density (CD) studies and refinements of data sets of normal resolution (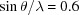
 Å^−1^ or 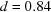
 Å) using scattering-factor databases, of which the GID is one.^1^

Scattering of H atoms is limited in reciprocal space owing to their comparably low scattering contribution and their missing core density. XRD therefore has limitations in locating their positions and displacements accurately (Cooper *et al.*, 2010 ▸), a situation that has already been improved considerably by scattering factor databases (Dittrich *et al.*, 2005 ▸) and Hirshfeld atom refinement (Jayatilaka & Dittrich, 2008 ▸; Capelli *et al.*, 2014 ▸), as comparisons with results from neutron diffraction have shown. These developments allow free refinement of hydrogen parameters but require good low-order data (Orben & Dittrich, 2014 ▸).

Several authors have proposed improved or alternative hydrogen treatment in XRD, mainly for CD work. With respect to positional parameters Hoser *et al.* (2009 ▸) recommend to only use low-order reflections from a high-resolution data set to determine the *X*—H directions, and then elongate to average neutron diffraction values (Allen & Bruno, 2010 ▸), whereas we advocate the use of calculated positions and *X*—H distances from the invariom database (Schürmann *et al.*, 2012 ▸), a procedure also applicable to data sets of low quality.

The *SHADE* (simple hydrogen anisotropic displacement estimator; Madsen, 2006 ▸) and *SHADE2* servers (Munshi *et al.*, 2008 ▸) can provide estimates of H-ADPs by combining a TLS (translation–libration–screw) fit (Schomaker & Trueblood, 1968 ▸) of the non-H atoms with average internal modes tabulated from neutron diffraction. The *SHADE2* server has established its usefulness in CD research.

Other ways to estimate ADPs for H atoms have been developed. Displacements can likewise be computed from spectroscopic data as implemented in the *SHADE3* server (Roversi & Destro, 2004 ▸; Madsen & Hoser, 2014 ▸). This idea was applied first by Hirshfeld & Hope (1980 ▸). One can also carry out theoretical optimizations of isolated molecular structures (Flaig *et al.*, 1998 ▸) or employ QM/MM cluster computations to retrieve the structure found in the crystal (Whitten & Spackman, 2006 ▸). Computed frequencies can subsequently be converted into internal atomic displacements, which are again combined with a TLS analysis^2^ after appropriate scaling  (Scott & Radom, 1996 ▸). Last but not least, full periodic computations, implemented in the *SHADE3* server, may also provide H-ADPs (Madsen *et al.*, 2013 ▸; Madsen & Hoser, 2014 ▸). However, all these approaches have disadvantages: estimates derived from diffraction data do not take into account temperature dependence of the internal contribution of atomic vibration;^3^ neutron data for the *SHADE2* approach are not available for rare bonding environments; theoretical studies require high computational costs and are thus unsuited for conventional structure determinations. This is why we introduce a new approach based on the invariom database, combined with a new freely available TLS analysis program.

Our approach relies on the geometry-optimized model compounds in the invariom database.^4^ It covers a wide range of chemical environments in organic chemistry (Dittrich *et al.*, 2013 ▸) and now also facilitates aspherical-atom refinements of coordination compounds (Dittrich *et al.*, 2015 ▸). Earlier work is here extended by providing functionality to estimate H-ADPs relying on the established empirical rules of partitioning electron density with invarioms. The rules already allow one to separate and reconstruct molecular EDDs from fragments and now also provide estimates of the internal modes of vibration of a particular chemical environment.

Estimation of H-ADPs thus allows further improvement of all those structures where chemical environments are covered by scattering factors of the GID. Moreover, estimated H-ADPs increase the choices in handling three common situations: (*a*) data of low quality can be better evaluated by reducing the number of refined parameters; (*b*) high-quality data of comparably low resolution are available; or (*c*) refinement of H-atom positions becomes an option when aspherical scattering factors and ADPs are kept fixed, thereby reaching better agreement with results from neutron diffraction and bond-length predictions of quantum chemistry.

Central to this work is the underlying development of a new segmented-body (Schomaker & Trueblood, 1998 ▸) TLS refinement program called *APD-Toolkit* (anisotropic proton displacement toolkit), which is introduced here.

## 2. Automatic segmented rigid-body analysis   

A simple approach that can provide information on the coupling between internal and external displacements is to assume segmented rigid-body motion. Our implementation analyzes the shape of all measured ADPs and determines how attached rigid groups should be added to the otherwise rigid body to best fit the observed ADPs. After internal and external contributions are estimated, a displacement model for H atoms is then generated by adding both contributions. The well known Fortran77 program for TLS fits *THMA14c* (Schomaker & Trueblood, 1998 ▸) is limited to 230 atoms in the asymmetric unit and can only handle up to seven manually defined attached rigid groups. These limitations were our motivation to develop a more flexible solution. Our program was developed to estimate the ADPs of H atoms and will be discussed next.

### 2.1. Workflow of the program   

The *APD-Toolkit* carries out the following steps:

(1) Determination of invariom names of all atoms.

(2) Calculation of internal displacement parameters from *GAUSSIAN* (Frisch *et al.*, 2013 ▸) output files and caching of results^5^ for subsequent applications.

(3) Transformation of internal ADPs to the crystal coordinate system.

(4) Calculation of the difference between observed and calculated internal ADPs for all non-H atoms to remove contamination of the TLS parameters with internal ADPs.

(5) Determination of a suitable segmentation model for the segmented rigid-body analysis.

(6) Computation of a physically meaningful set of TLS+ARG (attached rigid group) parameters describing ADP differences.

(7) Computation of external ADPs for all H atoms based on the TLS+ARG parameters and the atomic coordinates.

(8) Estimation of H-ADPs by adding internal and external contributions.

### 2.2. Automated rigid-body segmentation   

*APD-Toolkit* automatically analyzes the shape of non-H-atom ADPs to obtain a suitable segmentation model for a segmented rigid-body analysis. In contrast to similar procedures in protein refinement (Painter & Merritt, 2006 ▸) the method implemented analyzes the refined model to find a physically plausible segmentation model instead of finding the model that minimizes the *R*_1_(*F*) value.

**Figure 1:**
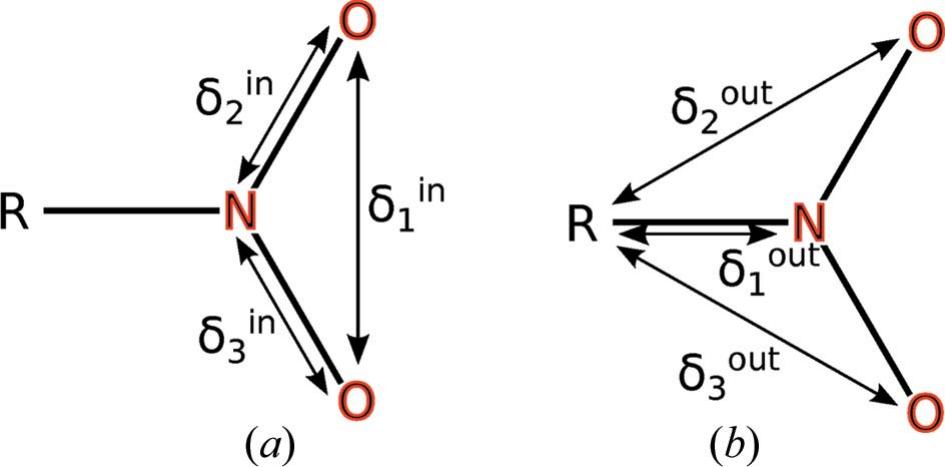
Illustration of the rigidity index: the NO_2_ group attached to a molecule R is considered rigid if the average of 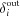 is twice as large as the average value of 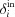.

The procedure works as follows. In a first step all single bonds in the molecule are flagged as potential axes for vibrations along a torsion angle (Blom & Haaland, 1985 ▸). For every potential axis the molecule is then separated into two parts that are connected only by one bond representing the rotation axis. The smaller of the two groups is considered to be the attached rigid group (Schomaker & Trueblood, 1998 ▸); the larger one is the rigid body. In a next step the difference of ADPs (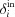) of a pair of bonded atoms in the direction of the connecting vector is computed for all atom pairs within the attached rigid group. In addition, the corresponding value (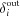) is determined for all atom pairs where one atom is part of the attached rigid group and the other atoms are part of the rigid body. For every potential rotation axis the ‘rigidity index’ Ω is then determined, as defined in equation (1) and illustrated in Fig. 1 ▸. If Ω is negative, the implied attached rigid group is accepted. The expression of Ω is purely empirical. The factor ∊ is used to control the weight between the rigidity of the ARG and the flexibility relative to the rest of the group. A value of 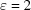 gave the most reasonable results in our studies.
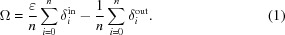



The groups are cross-referenced after all rigid groups are assigned to ensure that they all consist of at least eight atoms. Smaller groups are discarded, since they would not allow stable optimization.^6^ After a suitable segmentation model is found, a least-squares optimization is carried out to find the optimal TLS+ARG parameters.

The procedure is applied for each molecule in cases where the asymmetric unit contains more than one molecule. Adaptation of the procedure for disordered compounds and molecules on special positions is planned only for future versions.

## 3. Results and discussion   

### 3.1. Similarity of ADPs from TLS+ONIOM and TLS+INV^7^ estimates: similarity of *U*(2) from TLS+ONIOM and TLS+INV   

For initial validation, theoretical ADPs taken from the generalized invariom database were compared with those obtained from ONIOM computations (Svensson *et al.*, 1996 ▸) to assess the transferability of internal ADPs. Computations were performed with the B3LYP functional and the basis set combination 6-31G(d,p):3-21G, which has been shown to be a good compromise between computational requirements and the quality of results (Dittrich *et al.*, 2012 ▸). Internal ADPs are not compared directly since the internal parts of the ADPs encompass different parts of the overall displacements. Instead the values of internal displacements were combined with external displacements derived from TLS analysis, analogously to §3.3. For estimating internal ADPs a low-frequency cutoff value of 200 cm^−2^ was used (Madsen *et al.*, 2013 ▸).^8^

**Figure 2:**
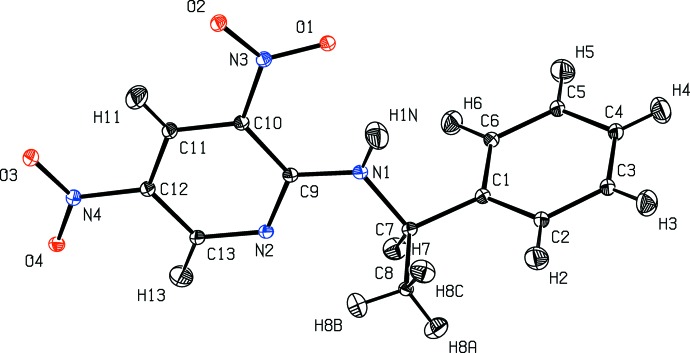
Structural model of methylbenzylaminodinitropyridine (MBADNP) at 20 K  (Cole *et al.*, 2002 ▸) with ADPs estimated with the TLS+INV approach.

Structural models of four test structures obtained by the TLS+INV approach are shown in Figs. 2 ▸–5 ▸ ▸ ▸.^9^

To quantitatively compare ADPs obtained by different methods a procedure proposed by Whitten and Spackman was employed (Whitten & Spackman, 2006 ▸; Munshi *et al.*, 2008 ▸). This procedure determines the spacial overlap of two sets of ADPs. It yields a value of the comparison parameter (*S*) of zero if both ADPs are identical and a value of 100 if the ADPs do not overlap at all. *S* is computed as 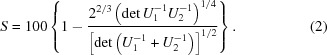



Tables 1 ▸–3 ▸ ▸ indicate that the agreement between the two methods depends on whether or not an H atom is involved in hydrogen bonding. In these cases the ONIOM estimate is more realistic since the bonding interactions, which are omitted in the TLS+INV approach, add forces to the H atoms that counteract vibrational movement. For those atoms not involved in hydrogen bonding the agreement is good, especially in cases where the asymmetric unit is described as one overall rigid body. This is supported by the very small discrepancies seen in the structures of MBADNP and xylitol. In these cases the non-hydrogen-bonded atoms have nearly identical ADPs. When the asymmetric unit content is more flexible or contains more than one molecule the agreement becomes less good, as evident in the structure of l-phenyl­alaninium hydrogen maleate. Since the TLS+INV approach does not include intermolecular interactions it predicts larger ADPs than the ONIOM model, which approximates these interactions. Slightly larger differences seen for methyl-group H atoms can also be explained by intermolecular interactions: while the rotational movement of the methyl group around a C—*X* (*X* not hydrogen) single bond usually has a discrete minimum for an isolated molecule, intermolecular interactions can lead to flattening of the potential, thus reducing the force required for rotating these groups.

Overall, the differences between the two methods are of the same order of magnitude as the differences seen between the estimated models and neutron diffraction derived models discussed below. We therefore argue that the TLS+INV method is an equivalent and easier to apply substitute for the computationally more demanding TLS+ONIOM approach. Empirical corrections for hydrogen bonding could be added at a later stage.

### 3.2. Temperature dependence of relative *U*_iso_ values   

**Figure 6:**
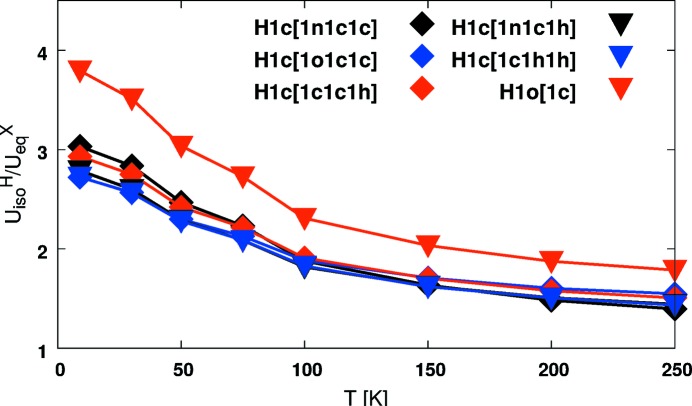
Temperature dependence of 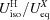 ratios obtained with the TLS+INV approach. The results are in very good agreement with those from our earlier study (Lübben *et al.*, 2014 ▸) reporting neutron and TLS+ONIOM results. Note: H atoms with the invariom name *H*1*c*[1*c*1*h*1*h*] are disordered and therefore appear larger when compared with the TLS+INV model. The H atom with the invariom name *H*1*o*[1*c*] is involved in hydrogen bonding, which is not accounted for in the TLS+INV model. Therefore, its 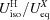 ratio is systematically larger.

Accounting for the measurement temperature when calculating the internal contributions to the ADPs avoids systematic errors that otherwise would affect data sets collected at low temperatures, especially below 100 K (Lübben *et al.*, 2014 ▸). That the temperature-dependent behavior is well reproduced in the TLS+INV approach introduced here is shown by comparing the temperature dependence of *U*_iso_ values obtained by the TLS+INV model with those determined from neutron diffraction studies and from ONIOM cluster computations in the same manner as in our earlier work (Fig. 6 ▸).

These results are in very good agreement with those of Lübben *et al.* (2014 ▸) and reproduce the temperature dependence. Additionally, the TLS+INV approach is able to estimate unbiased ADPs in cases where H atoms are disordered. Since the invariom approach relies on non-interacting mol­ecules in the gas phase, displacement parameters of H atoms involved in hydrogen bonding are less well estimated.

### 3.3. Comparison with results from neutron diffraction   

Estimated ADPs were compared with ADPs refined against neutron diffraction data to further validate the TLS+INV method. A set of four structures where both high-resolution X-ray data and neutron data are available were taken from the literature (Cole *et al.*, 2002 ▸; Woińska *et al.*, 2014 ▸; Şerb *et al.*, 2014 ▸; Madsen *et al.*, 2003 ▸). A scaling model was fitted to each neutron data set to bring both sets of ADPs onto the same scale (Blessing, 1995 ▸). This was achieved by computing the set of parameters 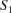
–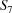 in equation (3) that minimize the difference between ADPs of equivalent atoms in both models. 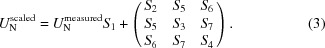



Modified TLS+INV results were then compared with those obtained with the *SHADE* server. One should note that the accuracy and absolute scale of H-ADPs remain unknown. While appropriate for atoms with similar mass, it has been shown that this scaling model does not yield accurate results when heavier atoms like iron are involved (Blessing, 1995 ▸). It is therefore reasonable to suspect that application of scaling parameters obtained by fitting against C and O atoms yields only rough estimates of hydrogen parameters. Absolute values of this comparison should therefore be interpreted with caution. Concerning this problem, the *SHADE* server benefits from error cancellation: since the internal ADPs are obtained from neutron diffraction studies, the ADPs are already scaled appropriately for comparison with neutron diffraction results and possible systematic errors could be obscured.

The *S* values listed in Tables 4 ▸–7 ▸ ▸ ▸ quantify differences between the respective methods of estimation and ADPs from models refined against neutron diffraction data. The estimation methods are thereby not compared directly; instead their agreement with experimental data is given.

Overall, one can clearly see that the *SHADE* server estimates are closer to the model refined against neutron diffraction data. However, the overall differences between all three models are of the same order of magnitude. It should also be noted that the values depend on the applied refinement model that was used prior to TLS analysis. A variation of about 0.5–0.7 in the average *S* values was observed when the invariom aspherical-atom model rather than the IAM model was used. Such variations do not appear to be systematic and can lead to smaller as well as larger *S* values. Therefore we conclude that the overall standard deviations of the *S* values must be of the same order of magnitude.

For the case of xylitol it is noteworthy that parameterization of the *SHADE* server was initiated with neutron data for this compound. Hence it is expected that the *SHADE* server performs especially well for this structure.

### 3.4. Usability   

The program *APD-Toolkit* was designed specifically to be easy to use. To demonstrate this a series of crystal structures were taken from the literature. The structures were re-refined with the invariom model and a subsequent TLS+INV treatment was applied. The TLS+INV application only requires one program call with output files from a previous refinement and no further input is needed. Currently *SHELXL*-style (Sheldrick, 2008 ▸) .res files, *XD*-style (http://xd.chem.buffalo.edu/ ) .res files, CIFs and PDB files (http://www.rcsb.org/) are supported. In Table 8 ▸ the effect of including the estimated H-ADPs on *R*_1_(*F*) was investigated. Respective refinement models use the same number of parameters.

Table 8 ▸ shows that the improvements in the *R*_1_(*F*) values are temperature dependent. We chose *R*_1_(*F*) (with units weights) for historic reasons and since unweighted *R*_2_(*F*) is not very meaningful. When non-hydrogen ADPs are large they increasingly deviate from the segmented rigid-body approximation, possibly because of anharmonic vibrational behavior (Zhurov *et al.*, 2011 ▸). Therefore TLS analysis may not provide accurate estimates of the lattice vibrations, and H-ADPs appear unreasonable large. On the other hand, when the measurement temperature is low and the refined ADPs are reasonable, the model including the estimated ADPs fits the measured data better, therefore also providing a useful indicator of data quality in the low-order region.

## 4. Conclusion and outlook   

The combination of segmented rigid-body analysis with information from geometry optimized model compounds allows one to rapidly estimate anisotropic hydrogen dis­place­ments from tabulated values in the TLS+INV approach introduced here. The invariom approach is thus being extended to predict not only aspherical scattering factors but also H-ADPs, all from one consistent model and notation. This is an important advantage over other scattering-factor databases.

The program *APD-Toolkit* provides an easy to use way of estimating these displacement parameters with an accuracy comparable to the TLS+ONIOM method without the need for extensive computations upon application. The software is a standalone alternative to the *SHADE* server, is freely available for download (http://ewald.ac.chemie.uni-goettingen.de/programs.html; https://github.com/jluebben/APD-toolkit) for various operating systems and can be easily adapted for other applications. The underlying TLS+ARG implementation can be combined with other software to generate segmented rigid-body models in an automatic fashion – without requiring specialized input-file formats or restrictions in system size.

## Figures and Tables

**Figure 3:**
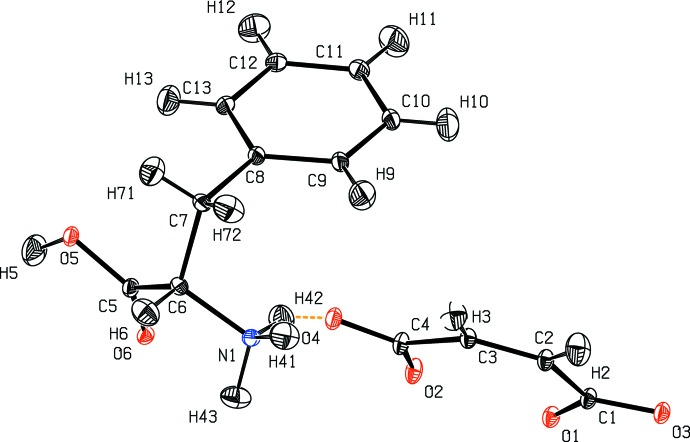
Structural model of l-phenylalaninium hydrogen maleate at 12 K (Woińska *et al.*, 2014 ▸) with ADPs estimated with the TLS+INV approach.

**Figure 4:**
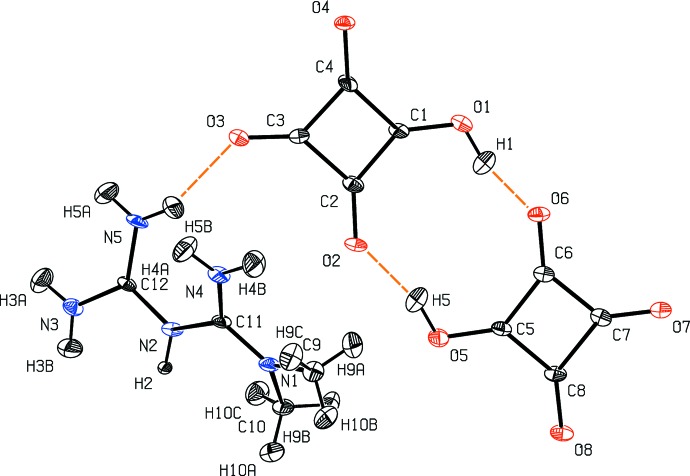
Structural model of dimethylbiguanidiniumbishydrogensquarate at 130 K  (Şerb *et al.*, 2014 ▸) with ADPs estimated with the TLS+INV approach.

**Figure 5:**
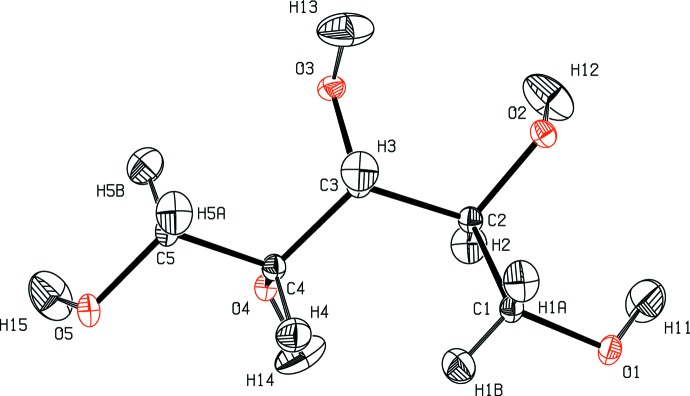
Structural model of xylitol at 122 K (Madsen *et al.*, 2003 ▸) with ADPs estimated with the TLS+INV approach.

**Table 1:** Comparison of TLS+INV derived ADPs with TLS+ONIOM derived ADPs of MBADNP

Label	*S*	Label	*S*
H11	0.31	H5	0.02
H13	0.07	H6	0.03
H1N	1.56	H7	0.02
H2	0.04	H8*A*	0.75
H3	0.03	H8*B*	0.52
H4	0.08	H8*C*	0.57
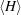	0.33		

**Table 2:** Comparison of TLS+INV derived ADPs with TLS+ONIOM derived ADPs of L-phenylalaninium hydrogen maleate

Label	*S*	Label	*S*
H10	1.52	H42	1.36
H11	1.41	H43	1.22
H12	1.43	H5	5.13
H13	1.97	H6	1.47
H2	0.64	H71	1.85
H3	1.57	H72	2.54
H41	4.15	H9	1.81
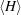	2.00		

**Table 3:** Comparison of TLS+INV derived ADPs with TLS+ONIOM derived ADPs of xylitol

Label	*S*	Label	*S*
H11	3.51	H1*B*	0.81
H12	9.58	H2	0.33
H13	3.84	H3	0.20
H14	13.60	H4	0.52
H15	10.52	H5*A*	0.37
H1*A*	0.80	H5*B*	0.57
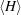	3.74		

**Table 4:** Comparison of TLS+INV (*S*_INV_) derived ADPs with *SHADE* (*S*_S_) ADPs for the example of MBADNP

Label	*S*_INV_	*S*_S_	Label	*S*_INV_	*S*_S_
H11	0.44	0.23	H5	0.75	0.28
H13	0.12	0.03	H6	1.17	0.27
H1N	1.35	0.39	H7	0.11	0.14
H2	0.17	0.09	H8*A*	1.76	1.30
H3	0.92	0.18	H8*B*	2.38	1.02
H4	0.17	0.14	H8*C*	2.21	0.90
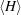	0.96	0.42			

**Table 5:** Comparison of TLS+INV (*S*_INV_) derived ADPs with *SHADE* (*S*_S_) ADPs for the example of L-phenylalaninium hydrogen maleate

Label	*S*_INV_	*S*_S_	Label	*S*_INV_	*S*_S_
H10	3.84	0.52	H42	4.80	0.70
H11	3.19	0.61	H43	3.91	1.08
H12	2.31	0.52	H5	3.82	1.33
H13	4.10	1.49	H6	2.94	0.67
H2	2.05	1.05	H71	13.68	5.71
H3	2.27	0.67	H72	1.90	0.38
H41	4.57	0.73	H9	3.22	0.90
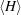	3.30	1.17			

The large discrepancy for atom H71 is most likely due to ill-determined displacement parameters in the neutron refinement, as becomes obvious from visual inspection.

**Table 6:** Comparison of TLS+INV (*S*_INV_) derived ADPs with *SHADE* (*S*_S_) ADPs for the example of dimethylbiguanidiniumbishydrogensquarate

Label	*S*_INV_	*S*_S_	Label	*S*_INV_	*S*_S_
H1	2.84	0.70	H4*B*	1.15	0.94
H10*A*	1.77	2.73	H5	4.04	0.37
H10*B*	2.15	3.97	H5*A*	0.73	0.06
H10*C*	1.60	2.42	H5*B*	0.70	0.13
H2	1.38	0.98	H9*A*	1.25	3.51
H3*A*	1.14	0.63	H9*B*	1.35	3.17
H3*B*	0.95	0.97	H9*C*	0.40	2.00
H4*A*	1.01	1.15			
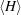	1.50	1.58			

**Table 7:** Comparison of TLS+INV (*S*_INV_) derived ADPs with *SHADE* (*S*_S_) ADPs for the example of xylitol

Label	*S*_INV_	*S*_S_	Label	*S*_INV_	*S*_S_
H11	3.55	0.58	H1*B*	2.45	0.74
H12	2.85	0.49	H2	0.62	0.55
H13	3.76	0.24	H3	0.07	0.09
H14	1.92	0.91	H4	0.28	0.10
H15	2.47	0.41	H5*A*	3.41	1.68
H1*A*	2.46	0.78	H5*B*	2.97	1.83
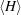	2.24	0.70			

**Table 8:** Temperature and resolution dependence of the improvements in the *R* value for a series of structure determinations

Structure code	*R*_TLS+INV_	*R*_riding_	δ_R_	Resolution (Å)	*T* (K)
hb6948 (Fadzillah *et al.*, 2012 ▸)	0.0272	0.0278	+0.007	0.73	100
zj2091 (Matos *et al.*, 2012 ▸)	0.0300	0.0307	+0.007	0.83	100
eg3095 (Tutughamiarso *et al.*, 2012 ▸)	0.0298	0.0301	+0.003	0.82	173
dt3014 (de Sousa *et al.*, 2012 ▸)	0.0533	0.0536	+0.003	0.80	173
yp3017 (Sonar *et al.*, 2012 ▸)	0.0529	0.0532	+0.003	0.83	90
fg3251 (Sowa *et al.*, 2012 ▸)	0.0580	0.0582	+0.002	0.81	100
bt5991 (Khalaji *et al.*, 2012 ▸)	0.0228	0.0230	+0.002	0.88	120
sh5011 (Madsen *et al.*, 2003 ▸)	0.0182	0.0182	+0.000	0.41	122
bi3042 (Liu *et al.*, 2012 ▸)	0.0474	0.0472	–0.002	0.73	153
fg3250 (Smith & Wermuth, 2012 ▸)	0.0316	0.0314	–0.002	0.81	293
fa3263 (Pérez *et al.*, 2012 ▸)	0.0441	0.0430	–0.011	0.77	293
fg3262 (Helliwell *et al.*, 2012 ▸)	0.0308	0.0280	–0.028	0.81	296

## Footnotes

1 Four scattering-factor databases currently exist: the ‘supramolecular-synthon based fragments approach’ (SBFA; Hathwar *et al.*, 2011 ▸), the ‘experimental library multipolar atom model’ (ELMAM2;  Zarychta *et al.*, 2007 ▸; Domagała *et al.*, 2012 ▸) (both based on high-resolution experiments), the ‘generalized invariom database’ (GID; Dittrich *et al.*, 2006 ▸, 2013 ▸) and the ‘University at Buffalo Databank’ (UBDB2011; Dominiak *et al.*, 2007 ▸; Jarzembska & Dominiak, 2012 ▸) (the latter two based on theoretical DFT computations). All four rely on the established Hansen/Coppens multipole model (Hansen & Coppens, 1978 ▸) and can successfully be used to improve the accuracy and precision of least-squares structure refinements.

2 In the context of this manuscript the term ‘TLS analysis’ is used for a post-refinement analysis of the atomic displacement parameters determined by the refinement program. The term ‘TLS refinement’ is not used to avoid confusion with procedures that apply a TLS model during the refinement process  (Merritt, 1999 ▸).

3 Neglecting temperature dependence of internal vibrational modes leads to systematic errors in the ADPs of H atoms  (Lübben *et al.*, 2014 ▸).

4 So far we have used the term ‘invariom database’ synonymously with ‘invariom scattering factor database’. Since now further functionality is being added, the invariom database becomes a database of model compounds from which several properties are derived.

5 Frequency information is extracted from all *GAUSSIAN* output files and stored in a database linking normal modes to the model compound including its geometry. This reduces the amount of data to store from several hundred gigabytes to about 100 MB. In a second step, when the method is applied to a structural model, the database is loaded and displacement parameters are calculated for all model compounds at the given temperature. The results are stored in a second database file now containing all internal ADPs at a certain temperature. The file is about 4 MB in size. During every subsequent application of the method it is checked whether the database already contains ADPs for the current temperature and ADPs are only recomputed if the method is applied at this temperature for the first time. Otherwise the cached results are used.

6 Theoretically two atoms, each providing six data points, should be sufficient since every attached rigid group adds seven parameters to the TLS model. The ADPs are, however, not completely independent of each other. Practical application shows a size of eight atoms to be a reasonable choice. If all potentially rigid groups consist of fewer than eight atoms, a rigid-body model is employed. Furthermore, TLS analysis fails if all atoms of a rigid group are placed on a conic section. Since this is likely for groups of six or fewer atoms, a group size of eight atoms avoids this problem in almost all cases. If a molecule consists of fewer than six atoms, the method cannot be applied and ADPs from the input file are used for the corresponding atoms.

7 INV stands for internal contributions computed from the generalized invariom database here.

8 Inaccuracies introduced in the estimation of internal displacements in the TLS+INV model are absorbed in the TLS part. We argue that the threshold separating internal and external displacements is arbitrary to some extent. Contributions to the displacements that are caused by vibrations with frequencies close to the threshold can be modeled by an internal and an external displacement model alike.

9 ONIOM computations for dimethylbiguanidiniumbishydrogensquarate did not converge and were therefore not included in the comparison.

## Appendix A Transformations and TLS fit

### A1. Coordinate transformation

The invariom database stores structural parameters like atomic positions and corresponding ADPs in a standardized format. These parameters need to be transformed to the crystal’s native coordinate system upon application.

Atomic positions are stored in an artificial crystal coordinate system in fractional coordinates. The artificial cell is cubic with a cell length of 30 Å.

ADPs are obtained from frequency computations carried out with *GAUSSIAN* and stored in a Cartesian coordinate system.

If *V* is the unit-cell volume, the matrix  is used to transform from fractional space to Cartesian space:

transforms from Cartesian to fractional systems:

If  is  with  Å and ° and  is  with the crystal’s cell parameters, the atomic position of an atom in the invariom database  in the crystal’s coordinate system  can be computed as

The matrix representation of an ADP in Cartesian space,

is transferred to the crystal’s coordinates system with

where

and

*a*, *b* and *c* are the cell constants of the crystal.

### A2. TLS+ARG fit

The TLS model describes the vibrational movement of a rigid atomic framework with 20 parameters in the form of the matrices *T*, *L* and *S*:

with

The six parameters of  can be expressed with *T*, *L*, *S* (Merritt, 1999 ▸; Schomaker & Trueblood, 1998 ▸) and the fractional coordinates  as

When  are the parameters obtained from structure refinement and *n* is the number of atoms, the set of parameters *T*, *L* and *S* are optimized to minimize

Each ARG rotating around the axis *t* adds seven parameters  to the  description, yielding the following expressions (Schomaker & Trueblood, 1998 ▸) if the atom at position *v* is part of the ARG:

with

and

where *P* is the shortest distance between *t* and the origin.

For *l* ARGs,  parameters are determined analogously to equation (16) by minimizing the expression
